# TSLC1和4.1B在非小细胞肺癌中的表达及临床意义

**DOI:** 10.3779/j.issn.1009-3419.2010.11.08

**Published:** 2010-11-20

**Authors:** 振华 王, 鲲鹏 杨, 旭广 王, 进 张, 德勋 郝, 占军 陈

**Affiliations:** 450014 郑州，郑州大学第二附属医院胸外科 Department of Thoracic Surgery, the Second Affiliated Hospital of Zhengzhou University, Zhengzhou 450014, China

**Keywords:** 肺肿瘤, 逆转录聚合酶链反应, TSLC1, 4.1B, Lung neoplasms, RT-PCR, TSLC1, 4.1B

## Abstract

**背景与目的:**

肺癌肿瘤抑制物1（tumor suppressor in lung cancer-1, TSLC1）属于细胞粘附分子中免疫球蛋白超家族成员，肺腺癌差异表达基因（differentially expressed in adenocarcinoma of the lung, 4.1B）属于NF2/ERM/4.1蛋白超家族成员之一，两者可能通过构建相邻细胞间稳定的粘附作用而抑制恶性肿瘤的发生。本研究通过检测TSLC1和4.1B在非小细胞肺癌中的表达及其与患者临床特征的关系，分析两种基因表达的相关性，以期为临床诊断与治疗提供理论基础。

**方法:**

采用RT-PCR的方法检测52例非小细胞肺癌组织以及52例相应癌旁正常肺组织中TSLC1和4.1B的表达。

**结果:**

TSLC1和4.1B在癌组织中的表达量明显低于癌旁正常肺组织（0.349±0.008 *vs* 0.555±0.010; 0.209±0.040 *vs* 0.721±0.071）（*P* < 0.01）。TSLC1和4.1B的表达与非小细胞肺癌的分化程度、TNM分期有关（*P* < 0.05），而与患者性别、年龄、病理分型无关（*P* > 0.05）。TSLC1与4.1B的表达呈正相关（*r*=0.471, *P* < 0.001）。

**结论:**

TSLC1和4.1B在非小细胞肺癌的发生中起抑制作用，两者可能通过级联反应共同参与了非小细胞肺癌的发生和发展。TSLC1和4.1B有望成为非小细胞肺癌基因诊断与治疗的靶点。

## Abstract (en)

**Background and objective:**

Tumor suppressor in lung cancer-1 (TSLC1) belongs to immunoglobulin superfamily of cell adhesion molecule and differentially expressed in adenocarcinoma of the lung (4.1B)belongs to NF2/ERM/4.1 protein superfamily. They may suppress carcinogenesis via construction of the adjacent cell adhesion stability. The aim of this study is to detect the expressions of TSLC1 and 4.1B in non-small cell lung cancer and the clinical pathological significances.

**Methods:**

The expressions of TSLC1 and 4.1B were detected by RT-PCR in 52 cases of non-small cell lung cancer and corresponding adjacent cancer lung tissues.

**Results:**

The expressions of TSLC1 and 4.1B in cancer tissues were significantly lower than that in adjacent cancer lung tissues (0.349±0.008 *vs* 0.555±0.010; 0.209±0.040 *vs* 0.721±0.071) (*P* < 0.01). The expressions of TSLC1 and 4.1B showed a significant correlation with cancer differentiation and TNM staging (*P* < 0.05), but not with gender, age and pathological type (*P* > 0.05). The expressions of TSLC1 and 4.1B were positively correlated (*r*=0.471, *P* < 0.001).

**Conclusion:**

Down-regulated expressions of TSLC1 and 4.1B in non-small cell lung cancer, both may participate in a cascade of non-small cell lung cancer occurrence and development. TSLC1 and 4.1B are promising targets for non-small cell lung cancer diagnosis and treatment.

非小细胞肺癌（non-small cell lung cancer, NSCLC）占肺癌的80%左右，肺癌的发生是多步骤、多阶段、多基因相互作用的结果，涉及多种癌基因的激活与抑癌基因的失活，其中抑癌基因的失活在近几年的研究中越来越受到重视^[1]^。肺癌肿瘤抑制物1（tumor suppressor in lung cancer-1, TSLC1）是Murakami^[2]^通过功能互补方法发现的一个肿瘤抑癌基因，该基因的突变与失活与人类多种肿瘤密切相关。肺腺癌差异表达基因（differentially expressed in adenocarcinoma of the lung, 4.1B）蛋白主要定位于细胞-细胞联结处，呈蜂窝状分布。研究^[3]^表明在脑膜瘤、乳腺癌、肾透明细胞癌和结肠癌等肿瘤组织中4.1B无论是RNA还是蛋白均出现表达下调或缺失现象。目前对上述两种基因的研究多集中于蛋白水平，且两者在NSCLC中表达的相关性国内报道尚少。本研究应用RT-PCR的方法从基因水平上检测TSLC1、4.1B在NSCLC中的表达，并分析两者的相关性及其与患者临床病理特征的关系，旨在为NSCLC的临床诊断、预测转移和分子治疗提供理论基础。

## 1 材料与方法

### 1.1 一般资料

所有标本均来自2009年12月-2010年4月间郑州大学第二附属医院胸外科手术切除并经病理证实为NSCLC的新鲜肿瘤组织52例及相应癌旁正常组织52例。其中男性33例，女性19例；年龄35岁-73岁，平均（55.12 ±8.61）岁；组织分型：鳞癌29例，腺癌23例；分化程度：高分化17例，中分化23例，低分化12例；TNM分期：Ⅰ期15例，Ⅱ期24例，Ⅲ期13例。所有病例术前均未进行放疗、化疗及其它针对肿瘤的生物学治疗。

### 1.2 方法

#### 1.2.1 RNA提取及RT-PCR扩增

取新鲜冰冻组织约100 mg加入Trizol 1 mL（按说明书操作）逐步提取总RNA，应用紫外分光光度计测定并计算所提RNA浓度。cDNA（complementry DNA）第一链合成体系为20 μL，取总RNA 8 μL、EasyScriptRT/RI Enzyme Mix 1 μL、Anchored Oligo(dT) 10 μL、2×ES Reaction Mix 1 μL，轻轻混匀，42 ℃孵育30 min，85℃加热5 min。取2 μL进行后续扩增，其余-80℃保存。基因*TSLC1*、*4.1B*及内参（β-actin）扩增应用Premier 5.0和Oligo 6.0软件设计引物（表 1），交北京赛百盛生物技术有限公司合成。PCR扩增反应体系：模板cDNA 2 μL、10×Easy Taq Buffer 5 μL、2.5 mmol/L dNTPs 4 μL、Easy Taq DNA Polymerase 1 μL、上、下游引物各1 μL加水至50 μL。PCR扩增循环条件：94 ℃预变性2 min，1个循环；94 ℃变性30 s，X ℃退火30 s，72 ℃延伸2 min，共35个循环；72 ℃总延伸6 min。最后4 ℃保存。

**1:** TSLC1、4.1B和内参*β*-actin引物列表 TSLC1、4.1B and *β*-actin primers list

Primer name		Sequence (5′-3′)	Annealing temperature (X)	Length
TSLC1	Sense	CAT AGT TGT CAT CCA GAA CCC AG	58 ℃	1 367 bp
	Antisense	GCT CCA GAC CTT GCC ATT TT		
4.1B	Sense	AGC ACA GCC CGC ATT CAC	56 ℃	145 bp
	Antisense	CTC CTC TTG TCG CTC ACG		
*β*-actin	Sense	CTG GGA CGA CAT GGA GAA AA	58 ℃	564 bp
	Antisense	AAG GAA GGC TGG AAG AGT GC		

#### 1.2.2 PCR产物结果判断及半定量分析

取PCR产物进行琼脂糖凝胶电泳，在紫外线投射仪下观察电泳条带，用D-140图像记录分析系统进行分析，目的基因mRNA的表达量以目的基因的DNA条带和β-actin的DNA条带灰度值比值计算。

### 1.3 统计学处理

所有数据用SPSS 11.5统计软件处理，数据均以Mean±SD表示，两组定量资料比较采用*t*检验，不满足*t*检验条件的采用*t*′检验。多组定量资料比较采用单因素方差分析（*one-way ANOVA*），组间比较采用*LSD-t*检验。指标间相关性用*Pearson*相关性分析。以*P* < 0.05为差异有统计学意义。

## 2 结果

### 2.1 TSLC1 mRNA与4.1B mRNA在NSCLC组织及相应癌旁正常组织中的表达

凝胶电泳显示，TSLC1 mRNA（1 367 bp）条带清晰无杂带扩增，目的基因片段产物与设计引物条带产物大小吻合，癌旁正常组织中条带亮度明显高于癌组织（图 1A）。4.1B mRNA（145 bp）条带清晰无杂带扩增，目的基因片段产物与设计引物条带产物大小吻合，癌旁正常组织中条带亮度明显高于癌组织中条带亮度（图 1B）。癌旁正常组织中TSLC1 mRNA与4.1B mRNA的相对表达量高于癌组织，差异有统计学意义（*P* < 0.01）（表 2）。

**1:**
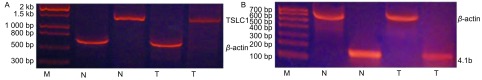
TSLC1 mRNA（A）和4.1B mRNA（B）PCR扩增产物凝胶电泳图。N为癌旁正常组织；T为癌组织；M为Mark。 Gel electrophoresis of PCR amplification products for TSLC1 mRNA (A) and 4.1B mRNA (B). N are from tumor adjacent tissues; T are from tumor tissues; M are from Mark.

**2:** TSLC1 mRNA与4.1B mRNA在NSCLC组织及相应癌旁正常组织中的表达 The expressions of TSLC1 mRNA and 4.1B mRNA in NSCLC and corresponding adjacent normal tissue

Tissues	*n*	Expression of TSLC1 mRNA				Expression of 4.1B mRNA		
		Relative expression level	*t*	*P*		Relative expression level	*t*	*P*
Adjacent normal tissues	52	0.555±0.010	15.886	< 0.01		0.721±0.071	45.068	< 0.01
Cancer tissues	52	0.349±0.008				0.209±0.040		

### 2.2 TSLC1 mRNA与4.1B mRNA的表达与NSCLC患者临床病理特征的关系

52例NSCLC组织中TSLC1 mRNA与4.1B mRNA的表达量在高、中分化组及TNM分期早期明显高于低分化组及TNM分期晚期，差异有统计学意义（*P* < 0.05），而与性别、年龄、组织分型无关（*P*>0.05）（表 3）。

**3:** TSLC1 mRNA与4.1B mRNA的表达与NSCLC患者临床特征的关系 The relationship between the expressions of TSLC1 mRNA and 4.1B mRNA and the clinical features

Clinical features	*n*	Expression of TSLC1 mRNA				Expression of 4.1B mRNA		
		Relative expression level	*t* (F)	*P*		Relative expression level	*t* (F)	*P*
Gender			-0.470	0.640			0.547	0.587
Male	33	0.346±0.059				0.211±0.040		
Female	19	0.354±0.051				0.205±0.041		
Age			0.136	0.892			-0.160	0.874
≥55y	32	0.350±0.051				0.208±0.038		
< 55y	20	0.347±0.063				0.210±0.045		
Pathology			1.455	0.152			0.582	0.563
Squamous cell carcinoma	29	0.359±0.057				0.212±0.039		
Adenocarcinoma	23	0.336±0.052				0.205±0.041		
Differentiation			5.029	0.010			11.032	< 0.001
Well	17	0.378±0.048				0.233±0.032		
Moderate	23	0.345±0.061				0.210±0.038		
Poor	12	0.317±0.032				0.173±0.029		
TNM stage			15.217	< 0.001			16.270	< 0.001
Ⅰ	15	0.394±0.044				0.244±0.031		
Ⅱ	24	0.348±0.050				0.205±0.034		
Ⅲ	13	0.301±0.032				0.176±0.027		

### 2.3 TSLC1 mRNA与4.1B mRNA在NSCLC组织中表达的相关性

*Pearson*相关性分析显示：肺癌组织中TSLC1 mRNA表达与4.1B mRNA表达之间呈正相关（*r*=0.471, *P* < 0.001）（图 2）。

**2:**
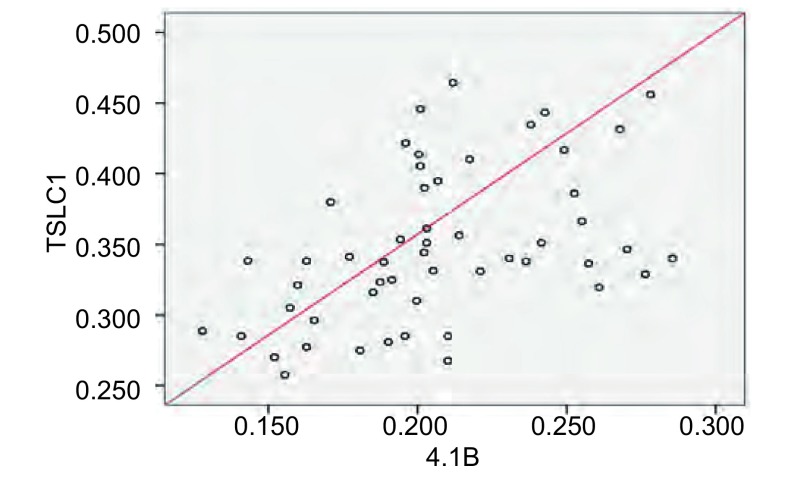
TSLC1 mRNA与4.1B mRNA在癌组织中表达水平的散点图 Scatter distribution of relative expressions of TSLC1 mRNA and 4.1B mRNA

## 3 讨论

TSLC1全长大约300 kb，定位于人类染色体11q23.2上，含10个外显子。翻译产物为含有442个氨基酸残基的跨膜糖蛋白。TSLC1属于细胞粘附分子中免疫球蛋白超家族成员编码细胞粘附分子（cell adhesion molecule, CAM），该分子是参与细胞与细胞之间相互作用的一种膜蛋白，除外周血淋巴细胞外人体绝大部分正常组织均表达TSLC1分子，它通过介导同种或异种细胞之间的相互粘附，在抑制恶性肿瘤的发生中起重要作用^[4]^。目前的研究^[5-7]^发现TSLC1的表达缺失或减少参与多种肿瘤的发生发展，包括乳腺癌、食管癌和宫颈癌。临床病理学研究分析显示TSLC1的失活在肿瘤晚期发生更频繁。Yang等^[8]^用免疫组化法发现TSLC1的表达与肿瘤分期、淋巴结受累、淋巴管渗透以及血管入侵呈负相关。因此我们推测TSLC1可能与肿瘤的生物学行为，包括侵袭和转移有关。本研究显示：TSLC1的表达与NSCLC的分化程度、TNM分期有关（*P* < 0.05），而与患者的性别、年龄以及病理分型无关（*P*>0.05），研究结果与国外现有文献^[9]^报道一致，说明TSLC1可能影响NSCLC的发生、发展和转移，TSLC1可以作为判断NSCLC恶性程度的一个重要的生物学指标。

4.1B属于4.1超家族蛋白，定位于人类染色体的18p11.3区域，4.1蛋白通过与肌动蛋白、血影蛋白等家族的蛋白质以及细胞膜蛋白的胞质区相互作用，维持细胞的正常形态和生理特性^[10]^。据Ohno等^[11]^报道4.1B在消化系统中可能不仅有维持其正常结构构建的作用而且有维持正常细胞增殖和粘附的作用，因此可抑制上皮细胞的恶性转化。Tran等^[12]^发现与癌旁肺组织相比，肺腺癌中4.1B mRNA表达水平明显降低，在所检测的10株细胞中有8株细胞4.1B mRNA表达缺失。本研究显示：与癌旁正常组织相比，4.1B蛋白在肺腺癌和肺鳞癌中的表达明显减少，并且还显示4.1B表达与NSCLC分化程度、TNM分期有关（*P* < 0.05），而与患者的性别、年龄以及病理分型无关（*P*>0.05），说明4.1B可能影响NSCLC的发生、发展和转移，进一步证实了*4.1B*是一种广泛存在的抑癌基因。

Yageta等^[13]^研究认为TSLC1通过4.1B与肌动蛋白骨架相连接，两种基因可能位于同一级联路径维持稳定的粘附结构。癌细胞中TSLC1或4.1B的缺失可能影响正常细胞粘附，导致肿瘤转移到邻近组织或末梢组织。TSLC1-4.1B级联反应也影响原发性脑脊膜瘤的发生发展^[14]^，这些都说明TSLC1和4.1B之间存在密切联系，我们用RT-PCR的方法研究了NSCLC中两种基因的表达及其相互关系，结果表明TSLC1与4.1B在NSCLC组织中的表达量明显低于相应癌旁正常肺组织（*P* < 0.01），TSLC1与4.1B在癌组织的表达呈正相关（*r*=0.471, *P* < 0.001）。

总之，TSLC1与4.1B在NSCLC组织表达下调或缺失均与NSCLC的分化程度及TNM分期有关，与患者的性别、年龄以及病理分型无关，并且二者具有相关性。TSLC1蛋白可能与4.1B蛋白共同参与NSCLC的发生、发展和转移。但我们目前仅是对其基因水平的表达情况进行了研究，要想确定其作为抑癌基因的功能还需要对上述两种基因是否具有生长抑制的功能及其作用的信号通路进行更深入的研究，从而进一步阐明TSLC1与4.1B在NSCLC发生发展过程中的作用机制，也将为NSCLC发病机制的研究提供新的依据，为NSCLC的临床诊断提供新的标志物，并有可能为肺癌的治疗提供新的靶点。
